# Effect of Music Therapy on Parent-Infant Bonding Among Infants Born Preterm: A Randomized Clinical Trial

**DOI:** 10.1001/jamanetworkopen.2023.15750

**Published:** 2023-05-26

**Authors:** Claire M. Ghetti, Tora Söderström Gaden, Łucja Bieleninik, Ingrid Kvestad, Jörg Assmus, Andreas Størksen Stordal, Luisa Fernanda Aristizabal Sanchez, Shmuel Arnon, Jeanette Dulsrud, Cochavit Elefant, Shulamit Epstein, Mark Ettenberger, Heidi Glosli, Ludwika Konieczna-Nowak, Marcela Lichtensztejn, Merethe Wolf Lindvall, Julie Mangersnes, Luz Dary Murcia Fernández, Catharina Janner Røed, Gladys Saá, Betty Van Roy, Bente Johanne Vederhus, Christian Gold

**Affiliations:** 1 GAMUT–The Grieg Academy Music Therapy Research Centre, The Grieg Academy–Department of Music, University of Bergen, Norway; 2 GAMUT–The Grieg Academy Music Therapy Research Centre, NORCE Norwegian Research Centre AS, Bergen, Norway; 3 Regional Centre for Child and Youth Mental Health and Child Welfare, NORCE Norwegian Research Centre AS, Bergen, Norway; 4 Institute of Psychology, University of Gdańsk, Gdańsk, Poland; 5 NORCE Energy, NORCE Norwegian Research Centre AS, Bergen, Norway; 6 Department of Mathematics, University of Bergen, Bergen, Norway; 7 Hospital Universitario Fundación Santa Fe de Bogotá, Bogotá, Colombia; 8 Clínica de la Mujer, Bogotá, Colombia; 9 Meir Medical Center, Kfar-Saba, Israel; 10 Sackler School of Medicine, Tel-Aviv University, Tel-Aviv, Israel; 11 Akershus University Hospital, Lørenskog, Norway; 12 University of Haifa, Haifa, Israel; 13 Oslo University Hospital, Oslo, Norway; 14 Music Therapy Department, The Karol Szymanowski Academy of Music in Katowice, Katowice, Poland; 15 Facultad de Ciencias de la Salud, Universidad de Ciencias Empresariales y Sociales, Buenos Aires, Argentina; 16 Department of Children and Youth Clinic, Haukeland University Hospital, Bergen, Norway; 17 Hospital General de Agudos Juan A. Fernández, Buenos Aires, Argentina; 18 Department of Pediatrics and Adolescent Health, Akershus University Hospital, Lørenskog, Norway; 19 Department of Clinical and Health Psychology, University of Vienna, Vienna, Austria

## Abstract

**Importance:**

Parent-infant bonding contributes to long-term infant health but may be disrupted by preterm birth.

**Objective:**

To determine if parent-led, infant-directed singing, supported by a music therapist and initiated in the neonatal intensive care unit (NICU), improves parent-infant bonding at 6 and 12 months.

**Design, Setting, and Participants:**

This randomized clinical trial was conducted in level III and IV NICUs in 5 countries between 2018 and 2022. Eligible participants were preterm infants (under 35 weeks’ gestation) and their parents. Follow-up was conducted across 12 months (as part of the LongSTEP study) at home or in clinics. Final follow-up was conducted at 12 months’ infant-corrected age. Data were analyzed from August 2022 to November 2022.

**Intervention:**

Participants randomized to music therapy (MT) plus standard care or standard care alone during NICU admission, or to MT plus standard care or standard care alone postdischarge, using computer-generated randomization (ratio 1:1, block sizes of 2 or 4 varying randomly), stratified by site (51 allocated to MT NICU, 53 to MT postdischarge, 52 to both, and 50 to neither). MT consisted of parent-led, infant-directed singing tailored to infant responses and supported by a music therapist 3 times per week throughout hospitalization or 7 sessions across 6 months’ postdischarge.

**Main Outcome and Measure:**

Primary outcome was mother-infant bonding at 6 months’ corrected age, measured by the Postpartum Bonding Questionnaire (PBQ), with follow-up at 12 months’ corrected age, and analyzed intention-to-treat as group differences.

**Results:**

Of 206 enrolled infants with 206 mothers (mean [SD] age, 33 [6] years) and 194 fathers (mean [SD] age, 36 [6] years) randomized at discharge, 196 (95.1%) completed assessments at 6 months and were analyzed. Estimated group effects for PBQ at 6 months’ corrected age were 0.55 (95% CI, −2.20 to 3.30; *P* = .70) for MT in the NICU, 1.02 (95% CI, −1.72 to 3.76; *P* = .47) for MT postdischarge, and −0.20 (95% CI, −4.03 to 3.63; *P* = .92) for the interaction (12 months: MT in NICU, 0.17; 95% CI, −2.71 to 3.05; *P* = .91; MT postdischarge, 1.78; 95% CI, −1.13 to 4.70; *P* = .24; interaction, −1.68; 95% CI, −5.77 to 2.41; *P* = .42). There were no clinically important between-group differences for secondary variables.

**Conclusions and Relevance:**

In this randomized clinical trial, parent-led, infant-directed singing did not have clinically important effects on mother-infant bonding, but was safe and well-accepted.

**Trial Registration:**

ClinicalTrials.gov Identifier: NCT03564184

## Key Points

**Question:**

What is the effect of parent-led, infant-directed singing—initiated in the neonatal intensive care unit with support from a music therapist and extending 6 months after discharge—on parent-infant bonding at 6 and 12 months’ infant-corrected age?

**Findings:**

In this randomized clinical trial involving 213 families, mothers engaging in parent-led, infant-directed singing reported similar parent-infant bonding at 6 and 12 months’ corrected age as mothers receiving standard care only.

**Meaning:**

These results suggest that, although safe and accepted by parents, parent-led, infant-directed singing was no more effective at improving mother-infant bonding than standard care.

## Teaser

This randomized clinical trial conducted in 5 countries examines the effect of parent-led, infant-directed singing on parent-infant bonding at 6 and 12 months.

## Introduction

Parent-infant bonding, an important contributor to quality of early parent-infant relationship and long-term infant health,^1^ may be disturbed or delayed with preterm birth.^2,3,4,5^ Bonding is influenced by parent-infant physical proximity, maternal emotional state, and infant communicative capacity,^4,6,7^ aspects that can be compromised in neonatal intensive care. Preterm infants demonstrate subtle and rapidly changing behavior states that can be difficult to interpret.^8,9^ Mismatched interactions between parents and infants can lead to parental frustration and overstimulation of the infant, which can be damaging for infant development.^8^ Music therapy focusing on parental singing contributes to parents’ sense of intimacy and communication with their preterm infants^10^ in ways that may support parent-infant bonding.

Prematurity is associated with poorer cognitive development, mental health, and quality of life for the child,^11^ and greater risk of stress, depression, and posttraumatic stress disorder for the parents.^3,12^ Music therapy in the neonatal context involves a music therapist partnering with parents to mindfully use music processes to promote stabilization and development of their infant, while supporting parental health and emergence of parent-infant relationship. Meta-analyses have demonstrated the short-term improvements music therapy can produce for infant physiological parameters and stress level and on maternal anxiety,^13,14^ although evidence of long-term effects is limited. Modest improvements in parent-premature infant bonding are demonstrated in pilot and small-scale music therapy studies,^15,16^ but the evidence is not consistent^17^ and definitive trials assessing longer-term effects are needed. Building on positive effects of parental singing on preterm infants and their parents,^18^ we conducted a randomized trial of parent-led, infant-directed singing to evaluate the effect of music therapy on longer-term mother-infant bonding, with secondary outcomes of infant development and parental well-being (anxiety, depression, stress).

## Methods

### Study Design

Longitudinal Study of Music Therapy’s Effectiveness for Premature Infants and Their Caregivers (LongSTEP) was designed as a 2 × 2 factorial, multinational, pragmatic randomized clinical trial (RCT) evaluating longer-term effects of music therapy (MT) in neonatal intensive care unit (NICU) and after discharge on preterm infants and their caregivers until 12 months’ infant corrected age.^19^ The factorial design enabled us to test main effects of both interventions (MT in NICU vs MT postdischarge), along with their interaction. Trial design, intervention protocol, and baseline characteristics of participants are published elsewhere.^19,20,21^ The original trial protocol is available in Supplement 1. Trial procedures and intervention were feasibility tested in 2 participating countries.^22,23^ The main trial received ethics approval from The Regional Committees for Medical and Health Research Ethics in Norway, supplemented by ethics approvals in each participating country. Participating parents provided written informed consent. This report followed the Consolidated Standards of Reporting Trials (CONSORT) reporting guidelines for RCTs.

### Participants

We recruited participants from 8 NICUs (7 level III and 1 level IV) in Argentina, Colombia, Israel, Norway, and Poland, countries where social systems enable continual parental presence during infant hospitalization. Preterm infants that were born at less than 35 weeks’ gestational age, were likely to be hospitalized a minimum 2 weeks from inclusion, and had been declared by NICU staff as medically stable to start MT (typically after 26 weeks’ postmenstrual age) were eligible for enrollment if they had parents who (1) provided written, site-specific informed consent, (2) were willing to engage in at least 2 of 3 MT sessions per week, (3) possessed sufficient understanding of the respective national language(s) to answer questionnaires and participate in MT, and (4) possessed sufficient mental capacity to complete the intervention and questionnaires.^19^

### Randomization and Masking

Following completion of written informed consent and baseline assessments, participants were randomized to MT plus standard care or standard care alone during NICU using a computer-generated randomization list with ratio 1:1, in block sizes of 2 or 4 varying randomly, stratified by site, using an online system (Sealed Envelope Ltd). Only the first-born infant of multiple pregnancies was included and randomized, while multiple-birth siblings received a corresponding intervention for ethical reasons. Before discharge home, participants were randomized a second time to postdischarge MT or standard care in a 1:1 ratio using the aforementioned procedures. It was not possible to mask participants, clinicians, or data collectors due to nature of the intervention and outcome measures.

### Intervention

Infants and parents in the NICU MT group received 3 individual MT sessions per week throughout hospitalization, with maximum 27 sessions, lasting approximately 30 minutes each (with time in active music making depending upon infant tolerance). MT consisted of parent-led, infant-directed singing; our use of the concept denoted a reciprocal process wherein infants through their communicative behavior actively direct parents’ use of voice, while parents then direct their singing to infant responses in the moment. Music therapists collaborated with parents to interpret subtle infant communicative cues and supported parents’ attuned vocal responses to such. Cautious use of sung or toned voice was tailored to infant postmenstrual age and readiness to receive stimulation, and matched to infant breathing patterns, facial expressions, and movements to promote quiet alert state or sleep, depending upon the infant’s needs. Parental voice was positioned as a resource,^24^ the MT process aimed to promote beneficial parent-infant mutual regulation, and included psychotherapeutic support of parents in alignment with trauma-preventive models.^25,26^ Participants in the postdischarge MT group received 7 individual MT sessions across the first 6 months after discharge from NICU, at home or at follow-up clinics, lasting approximately 45 minutes each. Principles of parent-led, infant-directed singing were expanded upon consistent with infant maturity, to include greater musical complexity and longer periods of interaction dependent upon infant tolerance. Parents were also encouraged to use principles of the intervention with their infants outside of MT sessions. Intervention was conducted by 11 NICU-specialized music therapists with master’s degrees in MT (or in terminal stage of degree [2 therapists]) who received training and supervision in the study intervention.^19,20^ Due to social isolation restrictions related to the COVID-19 pandemic, postdischarge MT sessions were offered virtually starting April 1, 2020 (eTable 1 in Supplement 2). Standard care varied across countries but included a range of medical, nursing, and social services in alignment with family-centered care.

### Outcomes

Our primary outcome was mother-infant bonding measured by the Postpartum Bonding Questionnaire (PBQ)^27^ total score (range, 0-125; 26 points or more indicating impaired bonding, 40 points or more indicating severe bonding disorder) (eAppendix in Supplement 2).^27^ Secondary outcomes were maternal depressive symptoms measured by the Edinburgh Postnatal Depression Scale (EPDS) (range, 0-30; high scores indicating worse symptoms)^28^; parental anxiety by the Generalized Anxiety Disorder tool (GAD-7) (range, 0-21; high scores indicating higher levels of anxiety)^29^; parental stress by the Parental Stress Scale (PSS) (range, 18-90; high scores indicating higher levels of stress)^30^; infant development by the Ages and Stages Questionnaire, third edition (ASQ-3)^31^ (range, 0-300; high scores indicating better development); infant socioemotional development by the Ages and Stages Questionnaire: Social-Emotional, second edition (ASQ:SE-2) (range, 0-345 for 6 months’ questionnaire, 0-405 for 12 months’ questionnaire; lower scores indicating better development)^32^; and rehospitalization during first year of life calculated as time from initial discharge until first rehospitalization. Details on secondary outcomes are available in the study protocol.^19^ Assessments were completed (1) before first randomization, (2) 1 to 2 days before discharge from the NICU, (3) 6 months’ corrected age, and (4) 12 months’ corrected age; exceptions for this schedule were made for the PSS, ASQ-3, and ASQ:SE-2, which were conducted only at 6 and 12 months’ corrected age. All severe adverse events were recorded by site investigators and reported to the core team, and further reported to the external data monitoring committee who assessed safety throughout the trial.

### Statistical Analysis

In the absence of a generally agreed minimal clinically important difference for the PBQ, we designed the trial to have 80% power to detect a difference of 4 points (corresponding to *d* = 0.5 if SD = 8).^19^ The target sample size of 250 was based on 2-sided 2.5% significance level (5%, Bonferroni corrected for 2 tests), clustered by country (ρ = 0.01) and with 20% attrition.^19^

Descriptive statistics characterizing the sample have been published elsewhere.^21^ We used an intention-to-treat approach, using all available data from participants as randomized. We examined intervention effects by testing group differences in PBQ total score by analysis of covariance (ANCOVA) with random intercept for site at 6 and at 12 months’ corrected age. As secondary analysis, we estimated the linear mixed effects model for PBQ total score at baseline and at 6 and 12 months’ corrected age depending on time, intervention, as well as their interaction with random intercept for individual and site. We carried out the same analyses with secondary outcomes. We conducted exploratory analyses of PBQ total scores for prespecified subgroups related to sex, hearing status at discharge, PBQ factor 1 impaired bonding score at baseline, parental socioeconomic status, average parental skin-to-skin care during NICU, duration of breastfeeding prior to 6-month corrected age, perceived emotional closeness during breastfeeding, and treatment per protocol.^19^ We completed analyses with statistics software R version 4.1.0 (R Project for Statistical Computing) and derived graphics using Matlab (Mathworks Inc).

## Results

**Figure 1.**
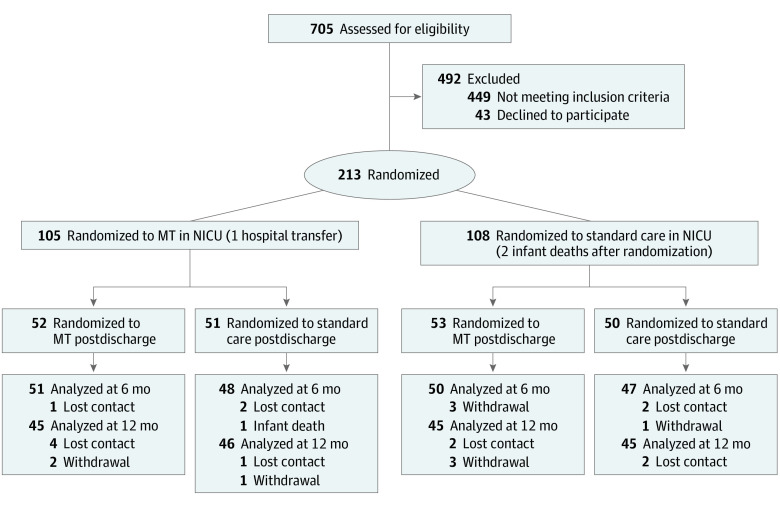
Study Flow Diagram The second randomization forms the basis for all numbers thereafter.

**Figure 2.**
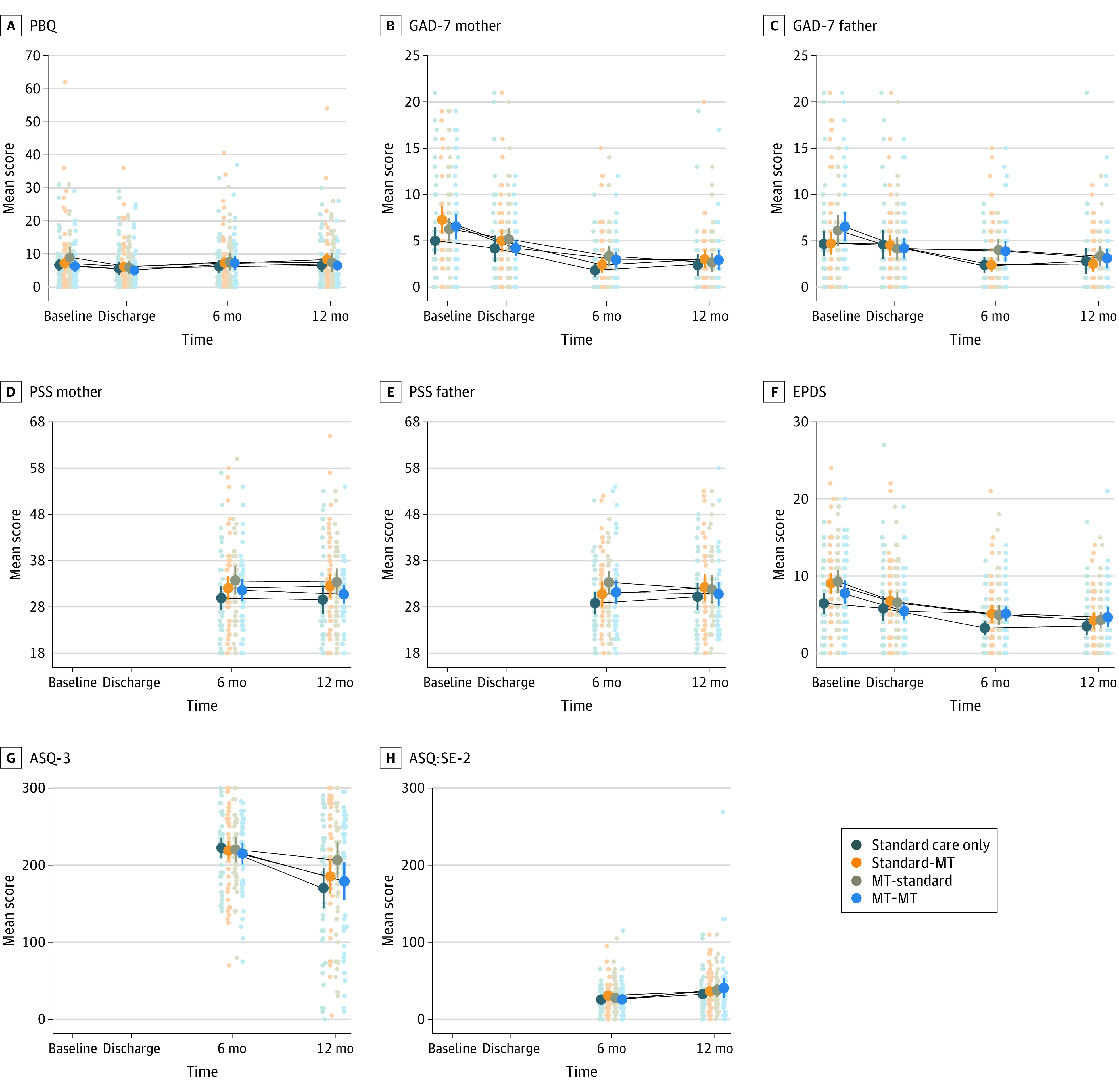
Mean Observed Values for Postpartum Bonding Questionnaire (PBQ) and Secondary Outcomes ASQ-3 indicates Ages and Stages Questionnaire, third edition; ASQ:SE-2, Ages and Stages Questionnaire: Social-Emotional, second edition; EPDS, Edinburgh Postnatal Depression Scale; GAD-7, Generalized Anxiety Disorder Assessment; MT, music therapy; PSS, Parental Stress Scale.

Of 213 infants randomized in NICU from August 2018 to April 2020,^21^ 206 infants (with 206 mothers and 194 fathers; mean [SD] maternal age, 33 [6] years; mean [SD] paternal age, 36 [6] years) were randomized at discharge to enter the present 2 × 2 factorial trial (103 female [50.0%]; mean [SD] gestational age, 30.5 [2.7] weeks; mean [SD] birth weight, 1400.5 [432.8] ounces; mean [SD] maternal postmenstrual age at enrollment, 33.1 [1.9] weeks). A total 196 of 206 (95.1%) mother-infant pairs completed assessments at 6 months’ corrected age, and 181 of 206 (87.9%) completed assessments at 12 months’ corrected age (Figure 1). Demographic and medical data were similar between study groups (Table 1).^21^ MT was provided by 11 music therapists (all female) (eTable 2 in Supplement 2). Estimated group effects for PBQ at main time point 6 months’ corrected age were 0.55 (95% CI, −2.20 to 3.30; *P* = .70) for MT at NICU, 1.02 (95% CI, −1.72 to 3.76; *P* = .47) for MT postdischarge, and −0.20 (95% CI, −4.03 to 3.63; *P* = .92) for the interaction (Table 2; Figure 2; LME results in eTable 3 in Supplement 2). Analysis of secondary variables revealed some nominal statistically significant differences for participants who received MT in 1 phase, although when considered within the context of the effect estimates for participants receiving MT in both phases, these differences do not appear to be clinically meaningful (eg, ASQ-3 score mean difference vs standard only: MT plus standard, 36.1; 95% CI, 0.8 to 71.5 vs MT both phases, 8.7; 95% CI, −27.7 to 45.0) (Table 3, Figure 2). There were no statistically significant differences for predefined subgroups, with no evidence of interaction detected prior to subgroup analysis (eTable 4 in Supplement 2).

**Table 1.** Baseline Characteristics Across 4 Study Arms^a^

Variable	MT/MT		MT/standard care		Standard care/MT		Standard care only	
	Participants, No.	Mean (SD)	Participants, No.	Mean (SD)	Participants, No.	Mean (SD)	Participants, No.	Mean (SD)
Infant sex (female), No. (%)	52	25 (48)	51	23 (45)	53	30 (57)	50	25 (50)
Birth weight, oz	52	1391.3 (420.9)	51	1364.0 (426.3)	52	1424.0 (464.1)	50	1422.9 (428.2)
Gestational age at birth, wk	52	30.2 (2.6)	51	30.4 (2.6)	53	30.48 (2.8)	50	30.9 (2.6)
Postmenstrual age at enrollment, y	52	32.8 (2.1)	51	33.3 (2.3)	53	33.1 (1.7)	50	33.1 (1.7)
Maternal age, y	51	34.1 (5.3)	51	32.1 (5.2)	51	32.2 (5.7)	50	33.4 (5.6)
Paternal age, y	49	36.5 (5.1)	48	35.2 (6.7)	50	34.5 (5.8)	47	36.6 (7.1)

Abbreviation: MT, music therapy.

^a^ For 206 participants who were randomized twice.

**Table 2.** Analysis of Covariance and Regression Results^a^

Result	6 mo infant-corrected age			12 mo infant-corrected age		
	Participants, No.	β (95% CI)	*P* value	Participants, No.	β (95% CI)	*P* value
**PBQ total score** ^b^						
MT at NICU	186	0.55 (−2.20 to 3.30)	.70	177	0.17 (−2.71 to 3.05)	.91
MT postdischarge		1.02 (−1.72 to 3.76)	.47		1.78 (−1.13 to 4.7)	.24
Interaction		−0.20 (−4.03 to 3.63)	.92		−1.68 (−5.77 to 2.41)	.42
**PBQ factor 1** ^b^						
MT at NICU	186	0.31 (−1.12 to 1.74)	.68	177	0.04 (−1.50 to 1.59)	.96
MT postdischarge		0.61 (−0.82 to 2.04)	.41		1.13 (−0.43 to 2.70)	.16
Interaction		−0.23 (−2.22 to 1.76)	.82		−0.87 (−3.06 to 1.32)	.44
**EPDS** ^b^						
MT at NICU	187	0.89 (−0.64 to 2.41)	.26	179	0.25 (−1.33 to 1.83)	.76
MT postdischarge		1.12 (−0.37 to 2.62)	.15		0.16 (−1.42 to 1.73)	.85
Interaction		−0.54 (−2.66 to 1.57)	.62		0.42 (−1.81 to 2.65)	.71
**GAD-7 mother** ^b^						
MT at NICU	187	1.34 (0.14 to 2.53)	.03	176	0.11 (−1.30 to 1.52)	.88
MT postdischarge		0.23 (−0.97 to 1.42)	.71		0.01 (−1.40 to 1.42)	.99
Interaction		−0.64 (−2.30 to 1.03)	.46		0.10 (−1.87 to 2.07)	.92
**GAD-7 father** ^b^						
MT at NICU	163	1.35 (−0.05 to 2.75)	.06	146	0.20 (−1.34 to 1.74)	.80
MT postdischarge		0.06 (−1.37 to 1.50)	.93		−0.48 (−2.02 to 1.06)	.55
Interaction		−0.28 (−2.27 to 1.71)	.78		0.23 (−1.91 to 2.37)	.84
**PSS mother** ^c^						
MT at NICU	181	3.84 (0.10 to 7.58)	.05	175	3.88 (0.17 to 7.59)	.04
MT postdischarge		2.16 (−1.56 to 5.88)	.26		3.06 (−0.72 to 6.83)	.12
Interaction		−4.30 (−9.53 to 0.92)	.11		−5.72 (−10.96 to −0.49)	.03
**PSS father** ^c^						
MT at NICU	162	4.50 (1.05 to 7.95)	.01	148	1.73 (−2.34 to 5.80)	.41
MT postdischarge		1.94 (−1.65 to 5.54)	.29		2.09 (−1.99 to 6.17)	.32
Interaction		−4.36 (−9.29 to 0.57)	.09		−3.27 (−8.93 to 2.38)	.26
**ASQ-3** ^c^						
MT at NICU	187	−1.85 (−20.23 to 16.52)	.84	179	38.46 (5.02 to 71.90)	.03
MT postdischarge		−3.98 (−22.25 to 14.28)	.67		17.28 (−15.95 to 50.50)	.31
Interaction		−1.38 (−27.15 to 24.38)	.92		−44.89 (−91.81 to 2.02)	.06
**ASQ:SE-2** ^c^						
MT at NICU	188	2.02 (−5.67 to 9.71)	.61	180	4.34 (−8.50 to 17.17)	.51
MT postdischarge		5.21 (−2.38 to 12.79)	.18		3.07 (−9.76 to 15.90)	.64
Interaction		−6.96 (−17.64 to 3.71)	.21		0.17 (−17.88 to 18.22)	.99

Abbreviations: ASQ-3, Ages and Stages Questionnaire, third edition; ASQ:SE-2, Ages and Stages Questionnaire: Social-Emotional, second edition; EPDS, Edinburgh Postnatal Depression Scale; GAD-7, Generalized Anxiety Disorder Assessment; PBQ, Postpartum Bonding Questionnaire; PSS, Parental Stress Scale.

^a^ Based on a linear mixed-effects model including variables mother-infant bonding, maternal depression, parental anxiety, parental stress, infant development, and infant social-emotional development with site as random effect.

^b^ Analysis of covariance.

^c^ Regression analysis.

**Table 3.** Observed Group Means and Mean Differences for All Outcomes

Group	6 mo infant-corrected age			12 mo infant-corrected age		
	Participants, No.	Mean (SD)	Mean difference vs standard only (95% CI)	Participants, No.	Mean (SD)	Mean difference vs standard only (95% CI)
**PBQ**						
MT/MT	50	7.3 (7.7)	1.1 (−1.9 to 4.0)	46	6.5 (5.9)	−0.1 (−2.9 to 2.7)
MT/standard	47	7.6 (9.1)	1.4 (−1.9 to 4.7)	46	7.4 (9.8)	0.8 (−2.8 to 4.4)
Standard/MT	49	7.3 (6.4)	1.1 (−1.6 to 3.9)	45	8.3 (7.0)	1.7 (−1.3 to 4.7)
Standard/standard	44	6.2 (6.7)	NA	44	6.6 (7.3)	NA
**PBQ factor 1**						
MT/MT	50	3.9 (4.0)	0.4 (−1.1 to 1.9)	46	3.8 (3.3)	0.1 (−1.4 to 1.6)
MT/standard	47	4.1 (4.5)	0.6 (−1.0 to 2.3)	46	4.0 (5.0)	0.3 (−1.6 to 2.2)
Standard/MT	49	4.0 (3.5)	0.5 (−0.9 to 1.9)	45	4.7 (3.8)	1.0 (−0.6 to 2.7)
Standard/standard	44	3.5 (3.4)	NA	44	3.7 (3.9)	NA
**EPDS**						
MT/MT	50	5.1 (3.3)	1.9 (0.5 to 3.2)	46	4.7 (4.4)	1.2 (−0.6 to 2.9)
MT/standard	46	4.9 (4.6)	1.7 (0 to 3.3)	46	4.3 (3.6)	0.8 (−0.8 to 2.3)
Standard/MT	49	5.1 (4.2)	1.9 (0.3 to 3.4)	45	4.2 (3.8)	0.7 (−0.9 to 2.3)
Standard/standard	44	3.2 (3.2)	NA	44	3.5 (3.8)	NA
**GAD-7 mother**						
MT/MT	49	2.9 (2.9)	1.1 (0 to 2.2)	46	2.9 (3.8)	0.5 (−1.1 to 2.2)
MT/standard	47	3.3 (3.7)	1.5 (0.2 to 2.8)	44	2.6 (3.6)	0.2 (−1.3 to 1.9)
Standard/MT	49	2.4 (3.0)	0.6 (−0.5 to 1.7)	45	3.0 (3.3)	0.6 (−0.9 to 2.2)
Standard/standard	44	1.8 (2.4)	NA	43	2.4 (3.9)	NA
**GAD-7 father**						
MT/MT	44	3.9 (3.9)	1.5 (0 to 2.9)	41	3.1 (3.5)	0.3 (−1.5 to 2.1)
MT/standard	42	4.0 (4.0)	1.6 (0.1 to 3.1)	37	3.4 (3.4)	0.6 (−1.3 to 2.4)
Standard/MT	39	2.4 (2.6)	0 (−1.2 to 1.2)	39	2.5 (2.9)	−0.3 (−2.0 to 1.4)
Standard/SC	41	2.4 (2.8)	NA	34	2.8 (4.2)	NA
**PSS mother**						
MT/MT	48	31.6 (8.3)	1.7 (−1.9 to 5.2)	45	30.7 (7.0)	1.1 (−2.6 to 4.9)
MT/SC	44	33.8 (10.3)	3.9 (−0.2 to 7.9)	46	33.4 (10.0)	3.8 (−0.4 to 8.1)
Standard/MT	45	32.1 (8.6)	2.2 (−1.5 to 5.8)	43	32.5 (8.6)	2.9 (−1.1 to 6.9)
Standard/standard	44	29.9 (8.8)	NA	41	29.6 (9.9)	NA
**PSS father**						
MT/MT	44	31.2 (8.7)	2.4 (−1.2 to 6.0)	40	30.8 (8.3)	0.6 (−3.4 to 4.5)
MT/standard	42	33.3 (8.2)	4.5 (0.9 to 8.0)	37	31.8 (9.6)	1.6 (−2.7 to 5.9)
Standard/MT	36	30.8 (8.3)	2.0 (−1.8 to 5.7)	37	32.2 (8.8)	2.0 (−2.1 to 6.2)
Standard/standard	40	28.8 (7.9)	NA	34	30.2 (8.7)	NA
**ASQ-3**						
MT/MT	48	214.8 (50.1)	−7.5 (−26.7 to 11.7)	46	178.9 (84.7)	8.7 (−27.7 to 45.0)
MT/standard	46	220.0 (54.7)	−2.3 (−22.8 to 18.2)	44	206.3 (77.9)	36.1 (0.8 to 71.5)
Standard/MT	48	218.5 (43.7)	−3.8 (−21.7 to 14.0)	45	185.2 (77.8)	15.0 (−20.1 to 50.2)
Standard/standard	45	222.3 (43.1)	NA	44	170.2 (88.6)	NA
**ASQ:SE-2**						
MT/MT	50	25.8 (20.7)	0.2 (−6.9 to 7.4)	46	40.6 (44.6)	7.6 (−7.2 to 22.6)
MT/standard	45	27.6 (19.1)	2.0 (−5.0 to 9.0)	45	37.7 (28.2)	4.7 (−6.2 to 15.6)
Standard/MT	48	30.7 (20.5)	5.1 (−2.0 to 12.4)	45	36.3 (22.7)	3.3 (−6.4 to 13.1)
Standard/standard	45	25.6 (14.0)	NA	44	33.0 (23.4)	NA

Abbreviations: ASQ-3, Ages and Stages Questionnaire, third edition; ASQ:SE-2, Ages and Stages Questionnaire: Social-Emotional, second edition; EPDS, Edinburgh Postnatal Depression Scale; GAD-7, Generalized Anxiety Disorder Assessment; MT, music therapy; NA, not applicable; PBQ, Postpartum Bonding Questionnaire; PSS, Parental Stress Scale.

We found no significant differences between groups in rate of rehospitalization at 6-month and 12-month corrected age (eFigure in Supplement 2). Four serious adverse events (infant death) occurred—2 during NICU hospitalization and 2 following discharge, all within the standard care group and not related to study procedures (eTable 5 in Supplement 2).

## Discussion

We used a multinational, pragmatic RCT to examine longer-term effects of parent-led, infant-directed singing supported by a music therapist on mother-infant bonding, parental well-being, and infant development from NICU hospitalization to 12 months’ corrected age. Parents had lower than anticipated levels of impaired bonding, anxiety, and depression at baseline. Although this parent-led form of music therapy was safe and well-accepted by parents, we found no clinically important effects on mother-infant bonding, nor on maternal anxiety, maternal depression, parental stress, or infant development.

Participants in all 4 groups demonstrated mean baseline PBQ scores well below cutoff points for impaired bonding, contrasting with previous research suggesting potential delay or disturbance following premature birth.^2,4^ Instead, our findings align with research suggesting that premature birth might stimulate bonding responses in parents living in countries with adequate resources.^33^ Parents in our sample are from countries with social policies enabling continuous parental presence in the NICU, and 92.0% (196 of 213) of participating mothers reported being present almost daily throughout hospitalization. Increased parental presence reduces parental stress and anxiety.^34^ Hence, standard care practices of the included NICUs may have supported parental mental health and bonding, resulting in low rates of impaired bonding. Furthermore, parents participating in this high-engagement study might not be representative of all parents in the participating NICUs.

On average, the infants in our sample were medically quite stable and more mature than those in similar studies (including mean gestational age at birth, mean postmenstrual age at enrollment, and proportions of infants with worst cranial ultrasound at discharge).^21,35^ In the protocol, infants were defined as eligible when the NICU medical team deemed them medically stable enough to start MT. Our data suggest that sites may have taken a conservative interpretation of this concept, leading to limited opportunities for intervention effects. Because research suggests that lower gestational age at birth is associated with greater parental stress and anxiety in NICU and across time,^3,4^ it may be that our participating parents with more stable infants experienced less distress, which may have affected our parental mental health and well-being outcomes.

Qualitative research from this trial suggests the intervention affects the parent-infant relation in ways not captured by the PBQ, namely by facilitating parental agency, parent-infant communication, and a sense of relationship with the infant in the postdischarge period.^36,37^ Observed effects exceeding 4 PBQ points in those with impaired baseline parental bonding as well as some other subgroups may warrant investigation in more targeted populations (eTable 4 in Supplement 2). Fathers in our study participated in approximately a quarter of MT sessions, and our findings provide preliminary indication that MT might reduce anxiety in fathers with elevated anxiety.

Our findings demonstrated neither a beneficial nor detrimental effect on infant development. Notably, although statistically significant for infants in the NICU MT group only, at 12 months’ corrected age ASQ-3 scores were higher in all MT groups, indicating improved development scores. However, our confidence in the NICU MT finding is low due to the wide variance in results and nonsignificant estimated effect for those receiving MT in both phases. A lack of conclusive results regarding infant development may be due to insufficient number of MT sessions, insufficiently sensitive outcome measures, or assessment occurring too early to detect the effect on neurodevelopment. A dose-dependent effect of creative MT on preterm infant brain development has been noted in previous research, when infants received nearly double the median MT sessions in the NICU than our study.^35^ Eighty-three percent of our participants received per-protocol MT in NICU, while only 60% received per protocol postdischarge. Although we aimed for parents to use skills learned in MT with their infants in other settings, and they reported doing so, it may be that a greater number of MT sessions would be required to demonstrate positive effects. Using American norms, all participants scored within the normal range for both general and social-emotional development.^31,32^ Although infants in all groups appeared to score poorer at 12 months than 6 months for general development, this trend did not differ between groups (Figure 2). Neurodevelopmental assessments show low stability in early childhood,^38^ and assessments later in childhood may render different results due to increased reliability.

### Strengths and Limitations

Strengths of the study included multicountry representation from a diversity of clinical settings, good per protocol intervention completion rates (83% NICU MT, 60% postdischarge MT), high study retention rates (95%), and use of a wide array of psychometrically strong measurement tools. Lack of adverse events or longer-term detrimental outcomes linked to the intervention and high intervention completion suggest that the treatment was safe, consistent with tenets of individualized developmental care,^34^ and acceptable to parents. As a pragmatic RCT, the intervention fit with typical care in a variety of clinical and posthospitalization contexts.^39^ We used a flexible protocol anchored in guiding principles that communicated the desired processes we posited would enable our intended mechanisms of change.^19,20^ Our treatment fidelity analysis suggests that the intervention was delivered with an acceptable level of consistency across sites and cultural contexts. Our findings contribute to the limited experimental evidence on fathers’ engagement in MT.

This study had several limitations, including a potential lack of sensitivity in the PBQ to detect improvements for mothers who report good mother-infant bonding at baseline, and the possibility of a floor effect where low scores on baseline PBQ precluded the ability to detect changes over time. In addition, we relied on parental self-report, which may have led to social desirability bias, particularly with the PBQ. We cannot exclude the possibility of contamination effects in the NICU phase, as 6 of our 8 participating NICUs had at least some number of shared rooms. We encouraged fathers to engage in MT sessions as we hoped doing so would contribute to infant outcomes and paternal well-being; however, fathers participated in only 25% of MT sessions in both phases. Although all music therapists were trained in neonatal care and in the study protocol, they had differing lengths of NICU experience. The onset of the COVID-19 pandemic in March 2020 affected our final recruitment and led to a small number of postdischarge MT sessions being held online (eTables 1 and 2 in Supplement 2). Although informal feedback from music therapists and families suggested that the intervention was successfully adapted to online formats, this may have impacted outcomes.

Our results suggest that risk of problematic mother-infant bonding may vary across cultural contexts and may not be an appropriate target of intervention for all preterm mothers.^33^ Based on our data, we suggest that research focus on the association of MT with the parent-infant relationship in vulnerable infants and families, fathers with severe anxiety, and mothers at risk for depression. Research assessing long-term effects of MT on health-promoting parental outcomes such as well-being, quality of life, self-efficacy, or parental agency, infant development at 5 years, and biomarkers associated with development is indicated. The contrast between our quantitative results and the qualitative findings of a cohort of the trial^36,37^ suggest that mixed methods research may offer a crucial means of integrating our understandings of what MT offers premature parents and infants.

## Conclusions

In this RCT, parent-led, infant-directed singing facilitated by a music therapist did not have clinically important effects on parental mental health or infant development. Parent-led music therapy supported by a music therapist was safe and well-accepted by parents. Building on qualitative findings^36,37^ suggesting that desirable outcomes for parent-infant relationships were not captured in the present RCT, we recommend exploring other aspects of the parent-infant relationship and targeting vulnerable infants and families. These findings are generalizable to other countries where social policies and practices enable continuous parental presence in the NICU.

## Notes

Supplement 1.
Trial Protocol and Statistical Analysis Plan

Supplement 2. **eTable 1.** Amendments to the Published Study Protocol **eTable 2.** Music Therapy Intervention Descriptives **eTable 3.** LME results for Primary Outcome and Secondary Parental Well-being Outcomes **eTable 4.** Subgroup Analysis for the Primary Outcome **eFigure.** Risk for Rehospitalization **eTable 5.** Severe Adverse Events **eAppendix.** Description of Primary Outcome Variable

Supplement 3.
Data Sharing Statement

## References

[1] Le Bas GA, Youssef GJ, Macdonald JA, . The role of antenatal and postnatal maternal bonding infant development: a systematic review and meta-analysis. Soc Dev. 2019;29:3-20. doi:10.1111/sode.12392

[2] Korja R, Latva R, Lehtonen L. The effects of preterm birth on mother-infant interaction and attachment during the infant’s first two years. Acta Obstet Gynecol Scand. 2012;91(2):164-173. doi:10.1111/j.1600-0412.2011.01304.x 22007730

[3] Brunson E, Thierry A, Ligier F, . Prevalences and predictive factors of maternal trauma through 18 months after premature birth: a longitudinal, observational and descriptive study. PLoS One. 2021;16(2):e0246758. doi:10.1371/journal.pone.0246758 33626102 PMC7904178

[4] Trumello C, Candelori C, Cofini M, . Mothers’ depression, anxiety, and mental representations after preterm birth: a study during the infant’s hospitalization in a neonatal intensive care unit. Front Public Health. 2018;6:359. doi:10.3389/fpubh.2018.00359 30581812 PMC6293875

[5] Kim AR, Tak YR, Shin YS, Yun EH, Park H-K, Lee HJ. Mothers’ perceptions of quality of family-centered care and environmental stressors in neonatal intensive care units: predictors of and relationships with psycho-emotional outcomes and postpartum attachment. Matern Child Health J. 2020;24(5):601-611. doi:10.1007/s10995-020-02876-9 31912379

[6] Slomian J, Honvo G, Emonts P, Reginster J-Y, Bruyère O. Consequences of maternal postpartum depression: a systematic review of maternal and infant outcomes. Womens Health (Lond). Published online April 29, 2019;15:1745506519844044. doi:10.1177/1745506519844044 31035856 PMC6492376

[7] Lutkiewicz K, Bieleninik Ł, Cieślak M, Bidzan M. Maternal-infant bonding and its relationships with maternal depressive symptoms, stress and anxiety in the early postpartum period in a Polish sample. Int J Environ Res Public Health. 2020;17(15):5427. doi:10.3390/ijerph17155427 32731490 PMC7432717

[8] Sansavini A, Zavagli V, Guarini A, Savini S, Alessandroni R, Faldella G. Dyadic co-regulation, affective intensity and infant’s development at 12 months: a comparison among extremely preterm and full-term dyads. Infant Behav Dev. 2015;40:29-40. doi:10.1016/j.infbeh.2015.03.005 26021805

[9] Bozzette M. A review of research on premature infant-mother interaction. Newborn Infant Nurs Rev. 2007;7(1):49-55. doi:10.1053/j.nainr.2006.12.002

[10] McLean E, McFerran KS, Thompson GA. Parents’ musical engagement with their baby in the neonatal unit to support emerging parental identity: a grounded theory study. J Neonatal Nurs. 2019;25(2):78-85. doi:10.1016/j.jnn.2018.09.005

[11] Chawanpaiboon S, Vogel JP, Moller A-B, . Global, regional, and national estimates of levels of preterm birth in 2014: a systematic review and modelling analysis. Lancet Glob Health. 2019;7(1):e37-e46. doi:10.1016/S2214-109X(18)30451-0 30389451 PMC6293055

[12] de Paula Eduardo JAF, de Rezende MG, Menezes PR, Del-Ben CM. Preterm birth as a risk factor for postpartum depression: a systematic review and meta-analysis. J Affect Disord. 2019;259:392-403. doi:10.1016/j.jad.2019.08.069 31470184

[13] Bieleninik Ł, Ghetti C, Gold C. Music therapy for preterm infants and their parents: a meta-analysis. Pediatrics. 2016;138(3):e20160971. doi:10.1542/peds.2016-0971 27561729

[14] Yue W, Han X, Luo J, Zeng Z, Yang M. Effect of music therapy on preterm infants in neonatal intensive care unit: Systematic review and meta-analysis of randomized controlled trials. J Adv Nurs. 2021;77(2):635-652. doi:10.1111/jan.14630 33200833

[15] Kehl SM, La Marca-Ghaemmaghami P, Haller M, . Creative music therapy with premature infants and their parents: a mixed-method pilot study on parents’ anxiety, stress and depressive symptoms and parent-infant attachment. Int J Environ Res Public Health. 2021;18(1):18265. doi:10.3390/ijerph18010265 33396496 PMC7795112

[16] Ettenberger M, Ardila YM. Music therapy song writing with mothers of preterm babies in the neonatal intensive care unit (NICU)—a mixed-methods pilot study. Arts Psychother. 2018;58:42-52. doi:10.1016/j.aip.2018.03.001

[17] Corrigan M, Keeler J, Miller H, Naylor C, Diaz A. Music therapy and family-integrated care in the NICU: using heartbeat-music interventions to promote mother-infant bonding. Adv Neonatal Care. 2022;22(5):E159-E168. doi:10.1097/ANC.0000000000000910 34138791

[18] Filippa M, Panza C, Ferrari F, . Systematic review of maternal voice interventions demonstrates increased stability in preterm infants. Acta Paediatr. 2017;106(8):1220-1229. doi:10.1111/apa.13832 28378337

[19] Ghetti C, Bieleninik Ł, Hysing M, . Longitudinal study of music therapy’s effectiveness for premature infants and their caregivers (LongSTEP): protocol for an international randomised trial. BMJ Open. 2019;9(8):e025062. doi:10.1136/bmjopen-2018-025062 PMC6731830 31481362

[20] Gaden TS, Ghetti C, Kvestad I, Gold C. The LongSTEP approach: theoretical framework and intervention protocol for using parent-driven infant-directed singing as resource-oriented music therapy. Nord J Music Ther. 2021;31(2):107-132. doi:10.1080/08098131.2021.1921014

[21] Gaden TS, Ghetti C, Kvestad I, . Short-term music therapy for families with preterm infants: a randomized trial. Pediatrics. 2022;149(2):e2021052797. doi:10.1542/peds.2021-052797 34988583

[22] Bieleninik Ł, Konieczna-Nowak L, Knapik-Szweda S, Kwaśniok J. Evaluating feasibility of the LongSTEP (Longitudinal study of music therapy’s effectiveness for premature infants and their caregivers) protocol with a Polish cohort. Nord J Music Ther. 2020;2020:1-23. doi:10.1080/08098131.2020.1781233

[23] Ghetti CM, Vederhus BJ, Gaden TS, . Longitudinal study of music therapy’s effectiveness for premature infants and their caregivers (LongSTEP): feasibility study with a Norwegian cohort. J Music Ther. 2021;58(2):201-240. doi:10.1093/jmt/thaa023 33448286

[24] Rolvsjord R. Resource-oriented music therapy in mental health care. Barcelona Publishers; 2010.

[25] Loewy J. NICU music therapy: song of kin as critical lullaby in research and practice. Ann N Y Acad Sci. 2015;1337(1):178-185. doi:10.1111/nyas.12648 25773633

[26] Stewart K. PATTERNS—a model for evaluating trauma in NICU music therapy: part 1—theory and design. Music Med. 2009;1(1):29-40. doi:10.1177/1943862109338370

[27] Brockington IF, Fraser C, Wilson D. The Postpartum Bonding Questionnaire: a validation. Arch Womens Ment Health. 2006;9:233-242. doi:10.1007/s00737-006-0132-1 16673041

[28] McBride HL, Wiens RM, McDonald MJ, Cox DW, Chan EK. The Edinburgh Postnatal Depression Scale (EPDS): a review of the reported validity evidence. In: Zumbo BD, Chan EK, eds. Validity and validation in social, behavioral, and health sciences. Springer International Publishing; 2014:157-174. doi:10.1007/978-3-319-07794-9_9

[29] Spitzer RL, Kroenke K, Williams JBW, Löwe B. A brief measure for assessing generalized anxiety disorder: the GAD-7. Arch Intern Med. 2006;166(10):1092-1097. doi:10.1001/archinte.166.10.1092 16717171

[30] Berry JO, Jones WH. The parental stress scale: initial psychometric evidence. J Soc Pers Relat. 1995;12(3):463-472. doi:10.1177/0265407595123009

[31] Squires J, Bricker D. Ages & Stages Questionnaires (ASQ-3). 3rd ed. Brookes Publishing Co; 2009.

[32] Squires J, Bricker D, Twombly E. Ages & Stages Questionnaires: Social-Emotional. 2nd ed. Brookes Publishing Co; 2015.

[33] Hoffenkamp HN, Tooten A, Hall RA, . The impact of premature childbirth on parental bonding. Evol Psychol. 2012;10(3):542-561. doi:10.1177/14747049120100031122947677

[34] O’Brien K, Robson K, Bracht M, ; FICare Study Group and FICare Parent Advisory Board. Effectiveness of Family Integrated Care in neonatal intensive care units on infant and parent outcomes: a multicentre, multinational, cluster-randomised controlled trial. Lancet Child Adolesc Health. 2018;2(4):245-254. doi:10.1016/S2352-4642(18)30039-7 30169298

[35] Haslbeck FB, Jakab A, Held U, Bassler D, Bucher H-U, Hagmann C. Creative music therapy to promote brain function and brain structure in preterm infants: a randomized controlled pilot study. Neuroimage Clin. 2020;25:102171. doi:10.1016/j.nicl.2020.102171 31972397 PMC6974781

[36] Epstein S, Elefant C, Ghetti C. Israeli parents’ lived experiences of music therapy with their preterm infants post-hospitalization. J Music Ther. 2022:59(3):239-268. doi:10.1093/jmt/thac006 35661217

[37] Epstein S, Elefant C, Arnon S, Ghetti C. Music therapy spanning from NICU to home: an interpretative phenomenological analysis of Israeli parents’ experiences in the LongSTEP Trial. Nord J Music Ther. Published online March 9, 2023. doi:10.1080/08098131.2023.2180773

[38] Kvestad I, Hysing M, Ranjitkar S, . The stability of the Bayley scales in early childhood and its relationship with future intellectual abilities in a low to middle income country. Early Hum Dev. 2022;170:105610. doi:10.1016/j.earlhumdev.2022.105610 35728398

[39] Loudon K, Treweek S, Sullivan F, Donnan P, Thorpe KE, Zwarenstein M. The PRECIS-2 tool: designing trials that are fit for purpose. BMJ. 2015;350:h2147. doi:10.1136/bmj.h2147 25956159

