# Supplementary material for: Effect of Music Therapy on Parent-Infant Bonding Among Infants Born Preterm: A Randomized Clinical Trial
Source: JAMA Netw Open. 2023 May 26;6(5):e2315750. doi: 10.1001/jamanetworkopen.2023.15750 (PMC10220519; doi:10.1001/jamanetworkopen.2023.15750)
Supplement: Supplement 2. — eTable 1. Amendments to the Published Study Protocol eTable 2. Music Therapy Intervention Descriptives eTable 3. LME results for Primary Outcome and Secondary Parental Well-being Outcomes eTable 4. Subgroup Analysis for the Primary Outcome eFigure. Risk for Rehospitalization eTable 5. Severe Adverse Events eAppendix. Description of Primary Outcome Variable [file jamanetwopen-e2315750-s002.pdf]

Supplemental Online Content

Ghetti CM, Gaden TS, Bieleninik Ł, et al. Effect of music therapy on parent-infant bonding among infants born preterm: a randomized clinical trial. *JAMA Netw Open.* 2023;6(5):e2315750. doi:10.1001/jamanetworkopen.2023.15750

eTable 1. Amendments to the Published Study Protocol

eTable 2. Music Therapy Intervention Descriptives

eTable 3. LME Results for Primary Outcome and Secondary Parental Well-being Outcomes

eTable 4. Subgroup Analysis for the Primary Outcome

eFigure 1. Risk for Rehospitalization

eTable 5. Severe Adverse Events

eAppendix. Description of Primary Outcome Variable

This supplemental material has been provided by the authors to give readers additional information about their work.

© 2023 Ghetti CM et al. *JAMA Network Open.*

eTable 1. Amendments to the published study protocol

Domain	Original wording	Revised to	Rationale
Delineation of “PBQ at baseline” sub-groups	“bonding at baseline (PBQ impaired bonding score ≥11 vs < 11)”	“bonding at baseline (PBQ impaired bonding score >11 vs ≤ 11)”<br><br>[Date of change 1 January 2020]	To correct a typographical error
Implementation of intervention: MT post-discharge from hospital	“[MT post-discharge sessions]…will occur at home, municipal child health centres or follow-up clinics…”	Addition: “If in-person visits are not possible due to restrictions accompanying the COVID-19 pandemic, MT sessions post-discharge will be offered via videocalls. Procedures for the videocalls will follow the steps provided in the original protocol, with the exception that the music therapist will generally listen and observe when the parent is singing, instead of singing or providing musical accompaniment. Sites should return to offering in-person sessions, once that option becomes available again.”<br><br>[Date of change 1 April 2020]	Social isolation restrictions related to the COVID-19 pandemic impacted all participating sites and precluded use of in-person MT sessions. On April 1, 2020, the study received approval from the overseeing ethics committee to incorporate procedures for virtual MT sessions.

eTable 2. Music therapy intervention descriptives

Variable	MT in NICU		MT Post discharge	
	N	*n* (%)	*N*	*n* (%)
Received per protocol MT	105	87 (83)	105	63 (60)
Received virtual MT sessions[a]	105	0 (0)	105	23 (22)
	N	*M* (*SD*) [min, max]	*N*	*M* (*SD*) [min, max]
Total MT sessions	105	9.98 (5.95) [0, 27]	105	4.73 (2.53) [0, 14[b]]
Session length	105	32.84 (8.14) [0, 50]	92	43.65 (10.82) [0, 60]
Mother present	105	8.39 (6.01) [0, 27]	92	4.74 (2.4) [0, 14]
Father present	103	2.15 (3.39) [0, 20]	92	1.13 (1.82) [0, 7]
Both parents present	104	2.73 (3.69) [0, 18]	92	1.55 (1.98) [0, 7]
Treatment delivery[c]	72	4.88 (0.92) [1, 6]	40	4.95 (1.05) [1, 6]

[a]Participants who received one or more MT sessions digitally due to restrictions related to the COVID-19 pandemic; total 52 MT sessions at 6 sites; 2.26 (1.09)[1, 4]

[b]This value represents a single exception where one participant received greater than protocol due to human error

[c]Mean composite treatment delivery scores for 39 randomly selected participants (high=6; with 4=sufficient adherence, determined *a priori*)

© 2023 Ghetti CM et al. *JAMA Network Open.*

eTable 3. LME results for primary outcome and secondary parental well-being outcomes

	PBQ		GAD7 (Mother)		GAD7 (Father)		EPDS	
	B (95%CI)	p-value	B (95%CI)	p-value	B (95%CI)	p-value	B (95%CI)	p-value
Time effect		0.98		<0.001		<0.001		<0.001
BL	1		1		1		1	
6 months	-0.52 (-3.6, 2.57)		-3.18 (-4.81, -1.55)		-2.27 (-4, -0.54)		-3.18 (-4.91, -1.45)	
12 months	-0.1 (-3.18, 2.98)		-2.63 (-4.27, -0.99)		-1.86 (-3.68, -0.04)		-2.93 (-4.66, -1.2)	
Group differences		0.88		0.08		0.002		0.020
MT at NICU								
Standard care	1		1		1		1	
Music therapy	2.3 (-0.68, 5.28)		1.26 (-0.32, 2.84)		1.47 (-0.19, 3.13)		2.82 (1.14, 4.49)	
MT after discharge								
Standard care	1		1		1		1	
Music therapy	0.54 (-2.45, 3.52)		2.26 (0.69, 3.83)		0.07 (-1.6, 1.74)		2.58 (0.92, 4.23)	
Group effect (2-way interactions)		0.34		0.26		0.87		0.11
Time * MT at NICU								
6 months * MT	-0.85 (-5.17, 3.47)		0.26 (-2.03, 2.55)		0.17 (-2.26, 2.61)		-1.17 (-3.6, 1.27)	
12 months * MT	-1.45 (-5.78, 2.88)		-0.99 (-3.31, 1.32)		-0.89 (-3.43, 1.65)		-2.08 (-4.51, 0.36)	
Time * MT after discharge								
6 months * MT	0.67 (-3.62, 4.97)		-1.68 (-3.95, 0.59)		-0.02 (-2.48, 2.45)		-0.72 (-3.12, 1.67)	
12 months * MT	1.21 (-3.13, 5.56)		-1.62 (-3.92, 0.68)		-0.36 (-2.89, 2.17)		-1.84 (-4.26, 0.59)	
MT at NICU * MT after discharge	-3.18 (-7.38, 1.02)		-1.98 (-4.19, 0.24)		0.33 (-2.03, 2.68)		-4.08 (-6.42, -1.73)	
3-ways interactions		0.86		0.61		0.95		0.09
6 months * MT * MT	1.63 (-4.4, 7.66)		1 (-2.19, 4.19)		-0.53 (-3.97, 2.91)		2.44 (-0.95, 5.82)	
12 months * MT * MT	0.52 (-5.59, 6.62)		1.61 (-1.63, 4.85)		-0.3 (-3.84, 3.23)		3.73 (0.32, 7.15)	

Abbreviations: CI, confidence interval; PBQ, Postpartum Bonding Questionnaire; GAD-7, Generalized Anxiety Disorder Assessment; EPDS, Edinburgh Postnatal Depression Scale

© 2023 Ghetti CM et al. *JAMA Network Open.*

eTable 4. Subgroup analysis for the primary outcome

		Music therapy		Standard care		Mean difference (95% CI)	
Characteristic	Subgroup	n	M (SD)	n	M (SD)		p[1]
a) Effects of music therapy during NICU							
Analysis scope	Intention-to-treat	99	7.45 (8.26)	93	6.79 (6.55)	0.66 (-1.46 to 2.77)	0.54
	Per-protocol[2]	84	7.99 (8.68)	93	6.79 (6.55)	1.2 (-1.1 to 3.5)	0.30
Sex	Male	51	8.17 (8.79)	45	6.39 (7.54)	1.79 (-1.52 to 5.1)	0.29
	Female	48	6.68 (7.67)	48	7.17 (5.52)	-0.49 (-3.2 to 2.22)	0.72
Gestational age	< 28 weeks	21	6.73 (9.03)	14	9.94 (10.03)	-3.21 (-10.05 to 3.62)	0.34
	≥ 28 to < 32 weeks	44	8.2 (8.73)	38	6.46 (6.18)	1.74 (-1.55 to 5.04)	0.30
	≥ 32 weeks	34	6.91 (7.24)	41	6.02 (5.14)	0.9 (-2.06 to 3.85)	0.55
Hearing status	Confirmed normal	65	8.95 (9.34)	61	7.31 (7.17)	1.64 (-1.29 to 4.57)	0.27
	Abnormal or not tested	34	4.58 (4.51)	32	5.81 (5.11)	-1.23 (-3.6 to 1.15)	0.31
Bonding	Impaired[3]	3	16 (8)	4	20 (10.39)	-4 (-21.91 to 13.91)	0.59
	Not impaired	96	7.18 (8.16)	89	6.2 (5.73)	0.98 (-1.05 to 3.02)	0.34
Socioeconomic status[4]	Lower	13	5.69 (7.03)	13	9.92 (8.63)	-4.23 (-10.61 to 2.15)	0.18
	Other	84	7.79 (8.51)	79	6.28 (6.1)	1.51 (-0.77 to 3.79)	0.19
Skin-to-skin care	≥ 4 days/week	73	8.05 (8.83)	69	7.33 (6.97)	0.72 (-1.92 to 3.35)	0.59
	< 4 days/week	26	5.76 (6.23)	24	5.23 (4.95)	0.53 (-2.66 to 3.72)	0.74
Breastfeeding	None	28	6.18 (6.09)	22	8.01 (8.85)	-1.83 (-6.32 to 2.65)	0.41
	< 3 months	20	6.22 (10.18)	17	6.13 (5.94)	0.09 (-5.41 to 5.58)	0.97
	≥ 3 months	51	8.63 (8.44)	54	6.5 (5.65)	2.13 (-0.68 to 4.93)	0.14
Emotional closeness during feeding	Consistently	61	5.23 (6.28)	69	6.22 (6.33)	-1 (-3.19 to 1.2)	0.37
	Sometimes	21	11.05 (8.26)	17	8.25 (6.92)	2.8 (-2.2 to 7.8)	0.26
	Rarely	11	13.88 (13.39)	5	9.8 (9.01)	4.07 (-8.42 to 16.57)	0.49
b) Effects of music therapy after discharge							
Analysis scope	Intention-to-treat	99	7.3 (7.03)	91	6.92 (8.01)	0.38 (-1.78 to 2.55)	0.73

© 2023 Ghetti CM et al. *JAMA Network Open.*

Characteristic	Subgroup	Music therapy		Standard care		Mean difference (95% CI)	p[1]
		n	M (SD)	n	M (SD)		
	Per-protocol[2]	63	7.9 (7.51)	91	6.92 (8.01)	0.99 (-1.51 to 3.49)	0.44
Sex	Male	47	7.26 (8)	49	7.4 (8.54)	-0.14 (-3.49 to 3.21)	0.93
	Female	52	7.33 (6.11)	42	6.35 (7.4)	0.98 (-1.85 to 3.81)	0.49
Gestational age	< 28 weeks	20	7.16 (7.14)	15	9.15 (12.02)	-1.99 (-9.25 to 5.26)	0.57
	≥ 28 to < 32 weeks	39	8.37 (8.21)	41	6.41 (7.22)	1.96 (-1.49 to 5.41)	0.26
	≥ 32 weeks	40	6.32 (5.62)	35	6.54 (6.79)	-0.23 (-3.12 to 2.67)	0.88
Hearing status	Confirmed normal	65	7.95 (7.82)	59	8.37 (9.11)	-0.42 (-3.45 to 2.62)	0.79
	Abnormal or not tested	34	6.05 (5.09)	32	4.24 (4.39)	1.81 (-0.52 to 4.15)	0.13
Bonding	Impaired[3]	3	11.67 (5.86)	7	17.29 (9.6)	-5.62 (-17.57 to 6.33)	0.30
	Not impaired	98	7.21 (7.01)	85	6.09 (7.24)	1.12 (-0.97 to 3.21)	0.29
Socioeconomic status[4]	Lower	13	7.85 (6.11)	13	7.77 (9.81)	0.08 (-6.61 to 6.76)	0.98
	Other	83	7.29 (7.3)	78	6.77 (7.73)	0.52 (-1.83 to 2.86)	0.66
Skin-to-skin care	≥ 4 days/week	72	7.75 (7.52)	68	7.63 (8.54)	0.12 (-2.57 to 2.82)	0.93
	< 4 days/week	27	6.09 (5.46)	23	4.81 (5.81)	1.28 (-1.95 to 4.51)	0.43
Breastfeeding	None	27	8.01 (7.46)	22	5.82 (7.5)	2.19 (-2.14 to 6.52)	0.31
	< 3 months	19	4.75 (6.02)	18	7.68 (10.3)	-2.94 (-8.67 to 2.79)	0.30
	≥ 3 months	53	7.85 (7.06)	51	7.12 (7.41)	0.73 (-2.08 to 3.55)	0.61
Emotional closeness during feeding	Consistently	67	6.18 (6.18)	63	5.3 (6.45)	0.89 (-1.31 to 3.08)	0.43
	Sometimes	20	10.11 (8.87)	17	9.29 (6.6)	0.82 (-4.36 to 6)	0.75
	Rarely	8	10.5 (7.52)	8	14.7 (15.62)	-4.2 (-17.85 to 9.44)	0.51

[1] All overall tests of subgroup effects were non-significant (not shown).

[2] Per-protocol analysis was defined as including those who received at least 6 MT sessions in the NICU and at least 5 MT sessions post-discharge, respectively.

[3] Impaired bonding was defined as a baseline PBQ impaired bonding score >11; for the post-discharge analysis, this pre-planned cutoff was replaced by ≥ 10 because there were otherwise too few cases to analyze.

[4] Proxy for parental socioeconomic status, where “lower” is defined as cases with a single mother or where any parent had not completed secondary school.

© 2023 Ghetti CM et al. *JAMA Network Open.*

eFigure 1. Risk for rehospitalization

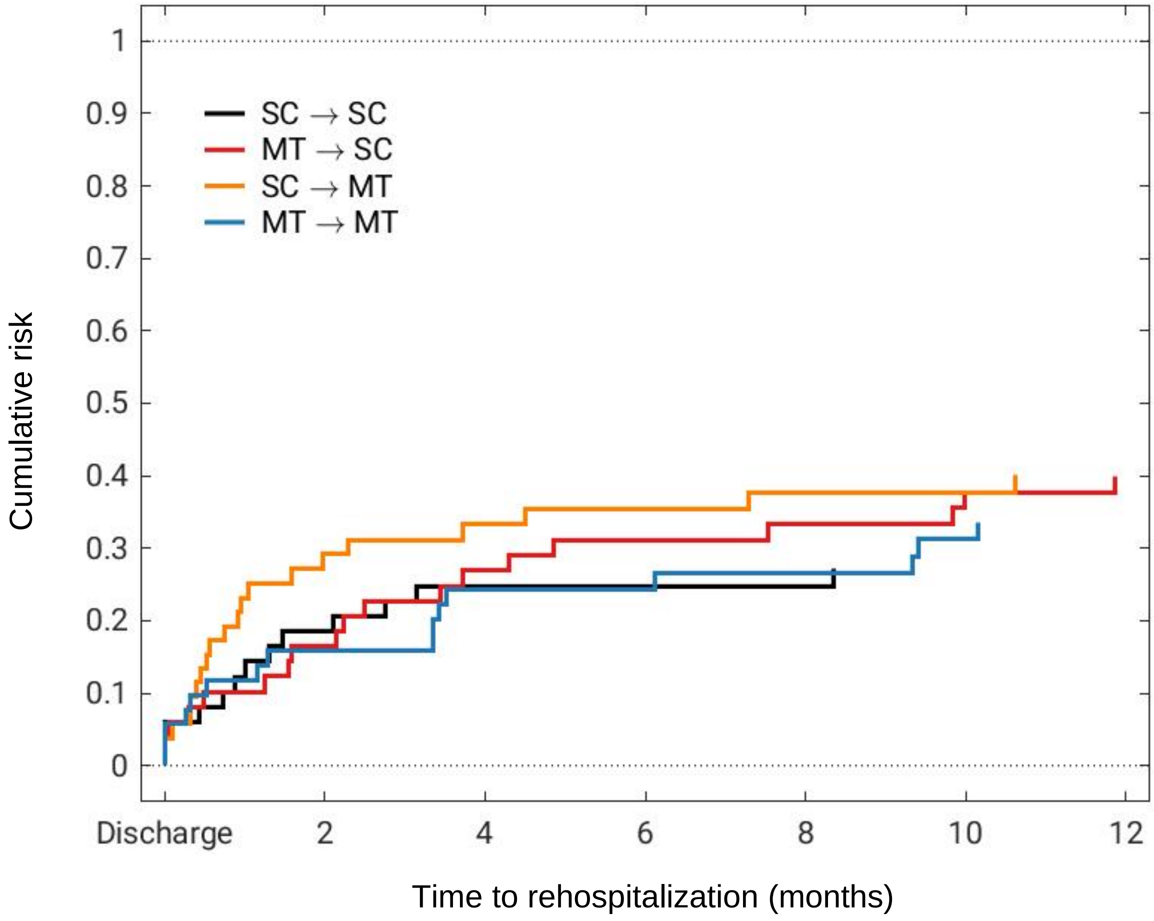

© 2023 Ghetti CM et al. *JAMA Network Open.*

eTable 5. Severe adverse events

Country	Timing of event	Nature of event	Likelihood of being related to study procedures
Argentina	During NICU stay	Infant death	Unrelated<br>(did not receive MT in NICU)
Poland	During NICU stay	Infant death	Unrelated<br>(did not receive MT in NICU)
Norway	Shortly after discharge to home	Infant death	Unrelated<br>(did not receive MT in NICU)
Colombia	Shortly after discharge to home	Infant death	Unlikely<br>(under SC at time of death)

© 2023 Ghetti CM et al. *JAMA Network Open.*

eAppendix. Description of primary outcome variable

The Postpartum Bonding Questionnaire (PBQ) evaluates mothers' feelings, experiences and attitudes toward their infant, using a 25-item self-report screening tool.[1,2] The PBQ is comprised of 4 factors, each with its own cut-off point: 1) general impaired bonding (12 items), 2) rejection and pathological anger (7 items), 3) anxiety about the infant (4 items), and 4) incipient abuse (2 items). Each item is rated on a 6-point Likert scale (each 0-5) with anchors “always” and “never,” with total scores ranging from 0-125 and higher scores indicating impaired bonding.

PBQ pre-specified subgroups for exploratory analyses: 1) sex; GA at birth (<28 weeks, 28 to <32 weeks, 32 to <35 weeks); 2) hearing status measured at discharge (normal vs. abnormal); 3) PBQ factor 1 impaired bonding score at baseline (>11 vs. ≤11); 4) proxy for parental socioeconomic status (education level, marital status); 5) average parental skin-to-skin care during NICU (4 or more days per week vs. fewer than 4 days per week); duration of breastfeeding prior to 6-month CA (none, fewer than 3 months, 3 months or greater); perceived emotional closeness during breastfeeding (consistently, sometimes, rarely); and treatment per protocol (≥6 NICU MT sessions; ≥5 post-discharge MT sessions).[3]

References

1 Brockington IF, Oates J, George S, et al. A screening questionnaire for mother-infant bonding disorders. *Arch Womens Ment Health.* 2001;3(4):133-40.

2 Brockington IF, Fraser C, Wilson D. The Postpartum Bonding Questionnaire: a validation. *Arch Womens Ment Health.* 2006;9:233-42. doi:10.1007/s00737-006-0132-1

3 Ghetti C, Bieleninik Ł, Hysing M, et al. Longitudinal Study of music Therapy's Effectiveness for Premature infants and their caregivers (LongSTEP): protocol for an international randomised trial. *BMJ Open.* 2019;9:1-16. doi:10.1136/bmjopen-2018-025062

© 2023 Ghetti CM et al. *JAMA Network Open.*
