# Supplementary material for: Effect of Music Therapy on Parent-Infant Bonding Among Infants Born Preterm: A Randomized Clinical Trial
Source: JAMA Netw Open. 2023 May 26;6(5):e2315750. doi: 10.1001/jamanetworkopen.2023.15750 (PMC10220519; doi:10.1001/jamanetworkopen.2023.15750)
Supplement: Supplement 3. — Data Sharing Statement [file jamanetwopen-e2315750-s003.pdf]

Data Sharing Statement

Ghetti. Effect of Music Therapy on Parent-Infant Bonding Among Infants Born Preterm: A Randomized Clinical Trial. *JAMA Netw Open*. Published May 26, 2023.
doi:10.1001/jamanetworkopen.2023.15750

Data
Data available: Yes
Data types: Deidentified participant data, Data dictionary
How to access data: Available through OSF through the following URL:
https://doi.org/10.17605/osf.io/smjka
When available: With publication

Supporting Documents
Document types: Statistical/analytic code
How to access documents: Available via OSF through URL:
https://doi.org/10.17605/osf.io/smjka)
When available: With publication

Additional Information
Who can access the data: Anyone requesting the data
Types of analyses: For any purpose
Mechanisms of data availability: Without investigator support
